# Theoretical and Experimental: The Synthetic and Anion-Binding Properties of Tripodal Salicylaldehyde Derivatives

**DOI:** 10.3390/s16050733

**Published:** 2016-05-19

**Authors:** Zhong-Jie Xu, Li-Rong Zhang

**Affiliations:** 1 School of Basic Medicine, Zhengzhou University, Zhengzhou 450052, China; xzj@xxmu.edu.cn; 2 Life Science and Technology College, Xinxiang Medical University, Xinxiang 453003, China

**Keywords:** molecular probe, tripodal compound, theoretical investigation, colorimetric binding, nano-material

## Abstract

A series of colorimetric anion probes **1**–**6** containing OH and NO_2_ groups were synthesized, and their recognition properties toward various anions were investigated by visual observation, ultraviolet–visible spectroscopy, fluorescence, ^1^H nuclear magnetic resonance titration spectra and theoretical investigation. Nanomaterials of three compounds **2**–**4** were prepared successfully. Four compounds **3**–**6** that contain electron-withdrawing substituents showed a high binding ability for AcO^−^. The host–guest complex formed through a 1:1 binding ratio, and color changes were detectable during the recognition process. Theoretical investigation analysis revealed that an intramolecular hydrogen bond existed in the structures of compounds and the roles of molecular frontier orbitals in molecular interplay. These studies suggested that this series of compounds could be used as colorimetric probes to detect of AcO^−^.

## 1. Introduction

Investigations of synthetic anion receptors have attracted considerable attention in the field of host-guest chemistry because of the important roles of anions in biomedicinal and chemical processes [1,2,3,4,5,6,7,8,9,10,11,12]. The design of these receptors has focused on the ability to recognize and sense the biologically important anions selectively. Water-soluble anions such as fluoride, chloride, bromide and phosphate are critical in a range of biological phenomena and are implicated in many disease states [13]. Acetate anions have unique chemical properties and can form the strongest hydrogen-bond interaction with hydrogen-bond donors because of the trigonal geometry and the high basicity. Much literature is available on selective receptor molecules for acetate anions [14,15,16]. Phosphorylated species play critical roles in a variety of fundamental processes and exist in many chemotherapeutic and antiviral drugs [17,18,19,20]. Phosphate that originates from the overuse of agricultural fertilizers can also lead to eutrophication in inland waterways [21]. To recognize and sense oxy-anions and phosphorylated biomolecules will become more important than other biologically functional anions [22,23,24].

**Scheme 1:**
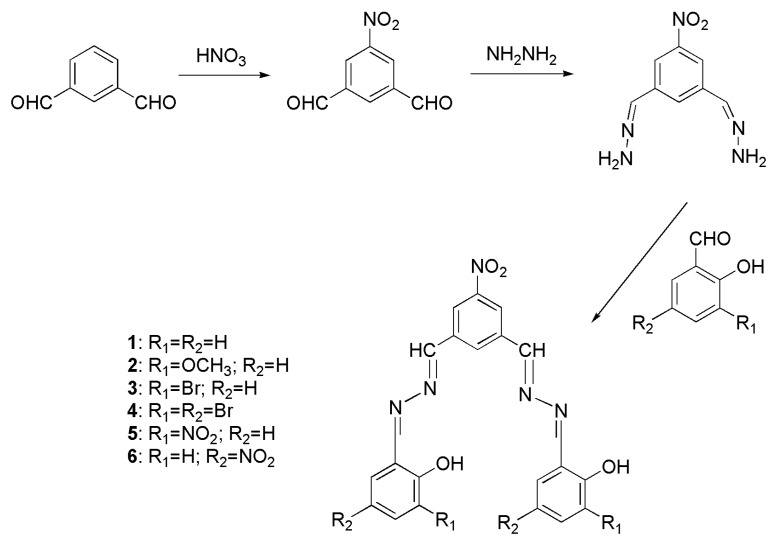
Synthesis of compounds **1**–**6**.

In many cases, a colorimetric receptor for special anionic species is of particular interest because of its simplicity and high sensitivity [25,26]. In particular, colorimetric-based sensing is especially attractive because it allows visible detection of analytes without requiring expensive equipment [27,28]. In general, these chemosensors are constructed according to a general receptor-chromophore general binomial, which involves the binding of a special anion substrate with receptor sites and a chromophore responsible for translating the receptor-anion association into an optical signal [29,30,31]. Color variation occurs when a charge-transfer complex is formed [32]. Therefore, it is necessary to develop a colorimetric anion receptor with high sensitivity and selectivity. We reasoned that a simple colorimetric anion receptor would be obtained by coupling the phenol group as a recognition site with a nitro chromophore as a signal group. Therefore, we synthesized a series of tripodal compounds that contain NO_2_ and a phenol group (Scheme 1). As expected, this series of compounds showed a strong binding ability for oxy-anions and a color change from yellow to orange occurred during the host–guest interaction. We also studied the effect of different substituents (*o*-OCH_3_, *o*-Br, *o,p*-Br, *o*-NO_2_ and *p*-NO_2_) to sensitivity and selectivity.

## 2. Experimental Section

### 2.1. Materials

Most of the starting materials were obtained commercially and all reagents and solvents used were of analytical grade. All anions, in the form of tetrabutylammonium salts (such as (*n*-C_4_H_9_)_4_NF, (*n*-C_4_H_9_)_4_NCl, (*n*-C_4_H_9_)_4_NBr, (*n*-C_4_H_9_)_4_NI, (*n*-C_4_H_9_)_4_NAcO, (*n*-C_4_H_9_)_4_NH_2_PO_4_), were purchased from Sigma-Aldrich Chemical Co. (Shanghai, China), and were stored in a desiccator under vacuum that contained self-indicating silica, and were used without any further purification. Tetra-*n*-butylammonium salts were dried for 24 h in vacuum with P_2_O_5_ at 333 K. Dimethyl sulfoxide (DMSO) was distilled *in vacuo* after being dried with CaH_2_.

### 2.2. Apparatus

Melting points were determined on a XT-4 binocular microscope (Beijing Tech Instrument Co., Beijing, China). C, H and N elemental analysis was achieved using Vanio-EL ^1^H nuclear magnetic resonance (NMR) (Bruker, Karlsruhe, Germany), and spectra were recorded on a Varian UNITY Plus-400 MHz Spectrometer (Agilent, Palo Alto, CA, USA). ESI-MS was performed with a MARINER apparatus (Agilent, Palo Alto, CA, USA). Ultraviolet (UV)–visible (vis) spectroscopy titrations were made using a Shimadzu UV2550 spectrophotometer (Shimadzu, Kyoto, Japan) at 298 K. Fluorometric titrations were performed on an Eclipse fluorescence spectrophotometer (Agilent, Palo Alto, CA, USA) at 298 K. Scanning electron microscopy (SEM) images were obtained by Quanta TM450 FEI with Au coating (Hillsboro, OR, USA). Optimized compound geometries were obtained used density functional theory at the B3LYP/3-21G level with Gaussian03 program (Pittsburgh, PA, USA). The affinity constant, *K*_s_, was obtained by the non-linear least squares calculation method for data fitting.

### 2.3. Synthesis

Compounds **1**–**6** were synthesized according to the route shown in Scheme 1.

#### 5-Nitro-1,3-dialdehydebenzene [33]

1, 3-Dialdehydebenzene (8.35 g, 35 mmol) was dissolved in sulfuric acid (28 mL). The solution was cooled to 1 °C and 100% HNO_3_ (4.5 mL, 0.11 mol) was added dropwise with stirring. The temperature did not exceed 1 °C. After the mixture had reacted for 4 h, the suspension was poured into ice water. The precipitate was filtered and recrystallized from ethanol. Yield: 89%. ^1^H-NMR (DMSO-*d_6_*): δ 10.12 (s, 2H, -CHO), 9.61 (s, 2H, ph-H), 9.05 (s, 1H, ph-H). Elemental analysis calcd. for C_8_H_5_NO_4_: C, 53.64; H, 2.81; N, 7.82; found: C, 53.90; H, 2.66; N, 8.17.

#### 5-Nitro-1,3-dimethylenehydrazine-benzene

5-Nitro-1,3-dialdehydebenzene (1 mmol, 180 mg) in dry ethanol (15 mL) was added to an ethanol (30 mL) solution containing hydrazine hydrate (80%, 0.5 mL) under stirring. Then, the mixture was heated under refluxing for 8 h and the yellow precipitate was separated by filtration. The solid was washed with diethyl ether and dried under vacuum. Yield: 84%. ^1^H-NMR (400 MHz, DMSO-*d*_6_, 298 K) δ 9.96 (s, 4H, NH_2_), 9.74 (s, 2H, ph-H), 9.18 (s, 1H, ph-H). Elemental analysis: calc. for C_8_H_9_N_5_O_2_: C, 46.38; H, 4.38; N, 33.80; found: C, 46.59; H, 4.26; N, 33.63.

Six compounds **1**–**6** were synthesized according to the following method. 5-Nitro-1,3-dimethylenehydrazine-benzene (1 mmol, 207 mg) was dissolved in dry ethanol (30 mL) and substituent salicylaldehyde (2 mmol) in dry ethanol (15 mL) was added to the solution under stirring. Then, the mixture was heated under refluxing for 8 h and the precipitate was separated by filtration. The solid was washed with diethyl ether, recrystallized with ethanol, and dried under vacuum.

Compound **1**: Yield: 73%. ^1^H-NMR (400 MHz, DMSO-*d*_6_, 298 K) δ 11.12 (s, 2H), 9.01 (s, 2H), 7.71–7.69 (dd, 2H), 7.57–7.54(dd, 4H) 7.41–7.40 (m, 4H), 6.97–6.70 (m, 3H). Elemental analysis: calc. for C_22_H_17_N_5_O_4_: C, 63.61; H, 4.12; N, 16.86; found: C, 63.38; H, 4.54; N, 16.63. ESI-MS (*m*/*z*): 414.2 [M − H]^−^.

Compound **2**: Yield: 77%. ^1^H-NMR (400 MHz, DMSO-*d*_6_, 298 K) δ 10.90 (s, 2H), 9.06–8.99 (m, 3H), 8.80 (d, 2H), 7.34–7.28(dd, 2H), 7.17–7.12(t, 2H), 6.94–6.89(dd, 2H), 3.83(s, 3H). Elemental analysis: calc. for C_24_H_21_N_5_O_6_: C, 60.63; H, 4.45; N, 14.73; found: C, 60.99; H, 4.31; N, 14.47. ESI-MS (*m*/*z*): 474.4 [M − H]^−^.

Compound **3**: Yield: 82%. ^1^H-NMR (400 MHz, DMSO-*d*_6_, 298 K) δ 11.21 (s, 1H), 11.14 (s, 1H), 9.03–8.94 (m, 5H), 8.83–8.79 (dd, 2H), 7.95–7.90 (dd, 2H), 7.55–7.53 (dd, 2H), 6.99–6.94 (dd, 2H). Elemental analysis: calc. for C_22_H_15_Br_2_N_5_O_4_: C, 46.10; H, 2.64; N, 12.22; found: C, 46.74; H, 2.28; N, 12.56. ESI-MS (*m*/*z*): 569.7 [M − H]^−^.

Compound **4**: Yield: 89%. ^1^H-NMR (400 MHz, DMSO-*d*_6_, 298 K) δ 12.31 (s, 2H), 9.13–9.05 (dd, 2H), 8.79 (d, 2H), 7.99–7.87 (m, 5H), 6.99–6.94 (dd, 2H). Elemental analysis: calc. for C_22_H_13_Br_4_N_5_O_4_: C, 36.15; H, 1.79; N, 9.58; found: C, 36.41; H, 1.68; N, 9.95. ESI-MS (*m*/*z*): 725.5 [M − H]^−^.

Compound **5**: Yield: 79%. ^1^H-NMR (400 MHz, DMSO-*d*_6_, 298 K) δ 12.37 (s, 2H), 9.22 (s, 2H), 9.18 (s, 1H), 9.14 (s, 1H), 8.82 (d, 2H), 8.15–8.05 (dd, 4H), 7.22–7.18 (t, 3H). Elemental analysis: calc. for C_22_H_15_N_7_O_8_: C, 52.28; H, 2.99; N, 19.40; found: C, 52.73; H, 3.15; N, 19.02. ESI-MS (*m*/*z*): 504.0 [M − H]^−^.

Compound **6**: Yield: 84%. ^1^H-NMR (400 MHz, DMSO-*d*_6_, 298 K) δ 12.19 (s, 2H), 9.06 (s, 2H), 9.01 (dd, 2H), 8.73 (s, 1H), 8.67 (d, 4H), 8.25–8.22 (dd, 2H), 7.15–7.12 (d, 2H). Elemental analysis: calc. for C_22_H_15_N_7_O_8_: C, 52.28; H, 2.99; N, 19.40; found: C, 52.73; H, 3.15; N, 19.02. ESI-MS (*m*/*z*): 504.3 [M − H]^−^.

### 2.4. Preparation of Nanomaterials

Nanomaterials were prepared by reprecipitation method [34,35]. The DMSO and the aqueous solution of hexadecyl trimethyl ammonium bromide (CTAB) were a good solvent and a poor solvent, respectively. The good compound-containing solvent (0.35 mL, 4 mmol·L^−1^) was poured into the poor solvent that contained CTAB (100 mL, 3 mmol·L^−1^). The mixture was centrifuged after 24 h. The resultant solid was washed with water and dried in vacuum.

## 3. Results and Discussion

### 3.1. SEM Images of Nanomaterials

**Figure 1:**
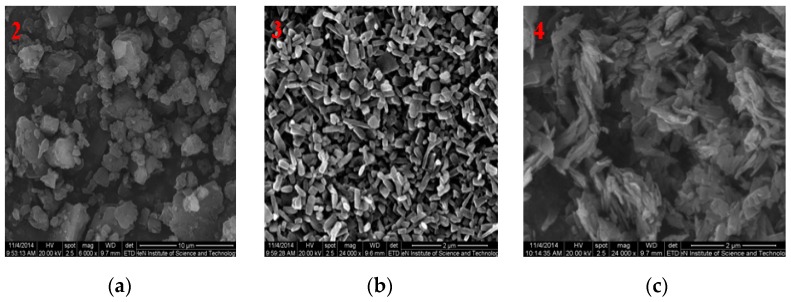
SEM images of compounds. (**a**) Compound **2**; (**b**) Compound **3**; (**c**) Compound **4**.

Only three of the attempted six nanomaterials (compound **2**–**4**) were prepared successfully. The SEM images were obtained using Quanta TM450 FEI with Au coating (Hillsboro, OR, USA) and were shown in Figure 1. Compound **2** was formed into a sheet. Compound **3** could be assembled into oval platelets over the entire compound. The flakiness was nanometer-wide according to the scale. For compound **4** that contained two bromine substituents, the platelets were stacked together in a flower-like shape. Although compounds **3** and **4** contained the same substituent (Br^−^), the SEM images were different because of the different numbers of bromine groups. Therefore, the SEM images were related with space configuration.

### 3.2. UV-Vis Titration

**Figure 2:**
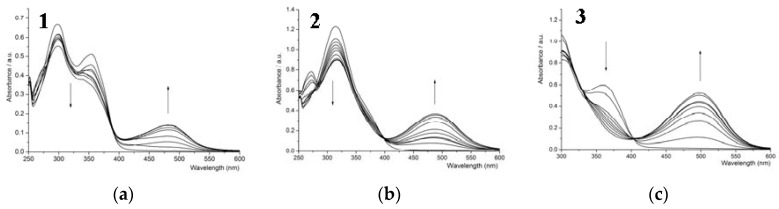

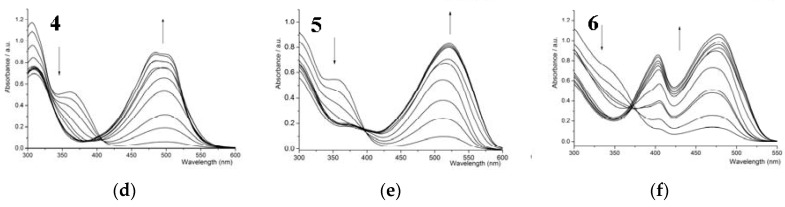
UV-Vis spectral changes of six compounds with acetate anion addition. [compound] = 4.0 × 10^−5^ mol·L^−1^; [acetate anion] = 0–1.6 × 10^−3^ mol·L^−1^. Arrows indicate the direction of increasing anion concentration. (**a**) Compound **1**; (**b**) Compound **2**; (**c**) Compound **3**; (**d**) Compound **4**; (**e**) Compound **5**; (**f**) Compound **6**.

**Figure 3:**
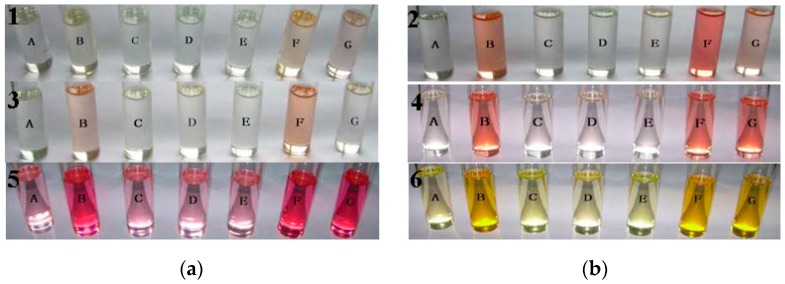
Color changes of six compounds with addition of various anions. (**A**) Blank; (**B**) F^−^; (**C**) Cl^−^; (**D**) Br^−^; (**E**) I^−^; (**F**) AcO^−^; (**G**) H_2_PO_4_^−^. (**a**) Compound **1**, **3** and **5**; (**b**) Compound **2**, **4** and **6**.

The binding abilities of six compounds to acetate anion were investigated using UV-Vis absorption spectra in DMSO at 298 K. The UV-Vis spectral changes of the six compounds were shown in Figure 2 during the titration with acetate anion. In the absence of acetate anion, compound **1** (4.0 × 10^−5^ mol·L^−1^ in DMSO) exhibited two obvious peaks centered at ~300 and 350 nm. With an increase in acetate concentration, the intensity of the above two absorption peaks strengthened and weakened, respectively. One new peak appeared centered at 475 nm. As a result, the red-shift phenomenon occurred after compound **1** interacted with acetate anion and the solution color changed from colorless to yellowish (Figure 3). Two isosbestic points appeared at 310 and 310 nm, which indicated the formation of stable complexation (**1**-AcO^−^). Analogous investigations were carried out on the other anions. The additions of H_2_PO_4_^−^ and F^−^ to compound **1** induced similar spectral change, which indicated that compound **1** also interacted with the above anions. However, Cl^−^, Br^−^ and I^−^ additions did not induce any spectral response, which indicated that compound **1** showed a very weak binding ability toward these anions and the binding ability could be ignored.

The acidity of this kind of compounds can be tuned by changing the electron property of the substituent on the ortho-, meta- or para-position according to resonance structure and the corresponding anion binding ability can also be changed correspondingly [36]. Therefore, **2** (*o*-OCH_3_), **3** (*o*-Br), **4** (*o*, *p*-Br), **5** (*o*-NO_2_) and **6** (*p*-NO_2_) were synthesized to investigate the effect of electron properties of the substituent on the host–guest interaction. As expected, the absorption spectra of **2**, **3**, **4**, **5** and **6** indeed exhibited various changes with an increase in acetate anion concentration (Figure 2) and were accompanied by color changes (Figure 3). Red-shift phenomena occurred to different degrees and one clear isosbestic point also appeared, which indicated that five compounds all interacted with acetate anion. Compared with compounds **1** and **2**, the red-shift effect of compounds **3** to **6** that contained electron-withdrawing groups was remarkably, which related to the substituent effect. In addition, compound **5** contained one nitro group, as well as compound **6**. However, the spectra of free compound and the host–guest complex were clear differently. The reason may be the different site of the nitro group. The above results indicated that the interacted mode and ability were related with the geometry structure.

### 3.3. Fluorescence

**Figure 4:**
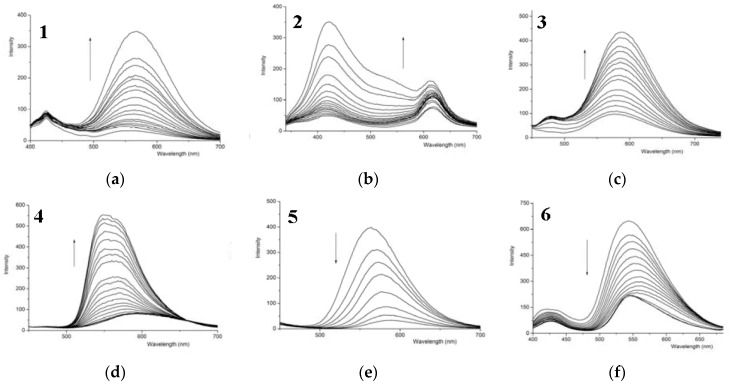
Fluorescence response of six compounds (4.0 × 10^−5^ mol·L^−1^) with acetate anion addition (0–2.0 × 10^−3^ mol·L^−1^). Arrows indicate the increase in direction of acetate concentration. (**a**) Compound **1**; (**b**) Compound **2**; (**c**) Compound **3**; (**d**) Compound **4**; (**e**) Compound **5**; (**f**) Compound **6**.

The photo physical responses of six compounds toward the additions of various anions tested were also investigated in DMSO solution. As shown in Figure 4, the fluorescence intensity of compounds **1**–**4** increased with the stepwise addition of acetate anion. Two possible mechanisms may explain the fluorescence enhancement: (1) inhibition of photo-induced electronic transfer (PET) [37] and (2) the guest binding-induced rigidity of the host molecule [38,39]. The oxygen atom of -OH could form an intramolecular hydrogen bond with near-hydrogen atoms (proven by theoretical investigation), which led to a PET and a decrease in fluorescence. However, the interaction between compounds and the acetate anion resulted in an inhibition of PET and an enhancement in fluorescent spectra intensity after acetate anion addition to a solution of compounds **1**–**4**. For two compounds **5** and **6**, the fluorescence intensity was quenched after acetate anion was added. The reason may be related to the strong electron withdrawing effect. The electron delocalization of two compounds **5** and **6** was strong and the rigidity structure was stable because of the electron-withdrawing effect of the nitro group. Therefore, free compounds **5** and **6** showed a strong fluorescence response before the acetate anion was added. After acetate anion addition, the strong rigidity structure was broken and the fluorescence intensity was decreased. Similar fluorescence spectral responses of compounds **1**–**6** were induced by the additions of other anions such as H_2_PO_4_^−^ and F^−^. Nevertheless, their fluorescence emission was insensitive to the additions of large quantities of other anions (Cl^−^, Br^−^ and I^−^), which indicated that the host-guest interactions were very weak and the anion binding ability could be ignored.

### 3.4. Binding Constant

Six compounds interacted with various anions in a 1:1 ratio according to the job–plot analysis. By the method of non-linear least squares calculation, the binding constants could be obtained and were listed in Table 1 based on the UV-Vis data [40,41,42]. From Table 1, four compounds that contained electron-withdrawing groups (**3**–**6**) all showed the strongest binding ability for AcO^−^ and a certain binding ability for F^−^ and H_2_PO_4_^−^ among the anions tested. However, two compounds (**1** and **2**) that contained electron-donating groups showed a strong binding ability for F^−^. The above results may be related to the space configuration. In addition, six compounds all showed a very weak binding ability for Cl^−^, Br^−^ and I^−^ could be ignored. π-π stacking may have existed between five nanomaterials and anions. For the acetate anion, the binding ability followed the order of: **6** > **5** > **4** > **3** > **1** > **2**. This order agreed with the ability of the electron-withdrawing group and the acidity of the six compounds. Compound **6** that contained an *m*-NO_2_ group showed the strongest binding ability for acetate anion among the six compounds. The anion binding ability of compound **5** that contained *o*-NO_2_ was weaker than that of compound **6** because of steric hindrance. In general, this series of compounds could be used as sensors to detect acetate anion.

### 3.5. ^1^HNMR Titration

**Figure 5:**
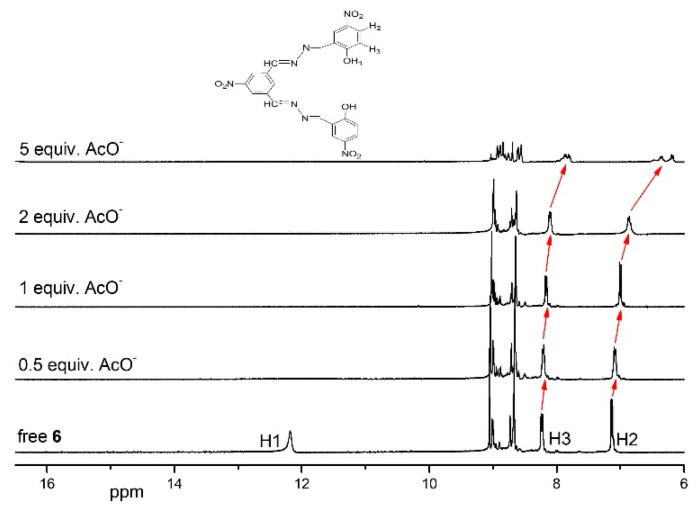
^1^H-NMR spectra of compound **6** in DMSO-*d*_6_ (1 × 10^−2^ mol·L^−1^) with addition of molar equivalent of AcO^−^.

To explain the interaction between the six compounds and the anions, as an example, ^1^H-NMR spectral changes were investigated upon AcO^−^ addition as its tetrabutylammonium salt to the DMSO-*d*_6_ solution of compound **6** (1 × 10^−2^ mol·L^−1^). Figure 5 showed that the peak at 12.19 ppm, which was assigned to −OH, broadened and thoroughly disappeared upon the addition of different equivalents of AcO^−^, which indicated that the strong hydrogen-bonding interactions occurred between compound **6** and the AcO^−^ ion [43]. In the nitro-phenol moiety, the protons (H2: 7.15–7.12 ppm; H3: 8.25–8.22 ppm) were overlapped when the anion was absent. With increase in AcO^−^ ion, the proton peaks of phenol all split and shifted in the upfield direction because of the deshielding effect on the nitrophenol moiety. When the AcO^−^ ion was added, the binding site (–OH group) interacted with AcO^−^ to form a shielding effect. Therefore, the proton peak of phenol moved downfield and disappeared. The non-interacted site experienced a deshielding effect and shifted in the upfield direction. The above results indicated that the synthesized compounds interacted with acetate anion through hydrogen-bonding, which was reversible in the host-guest interaction.

### 3.6. Theoretical Investigation

**Figure 6:**
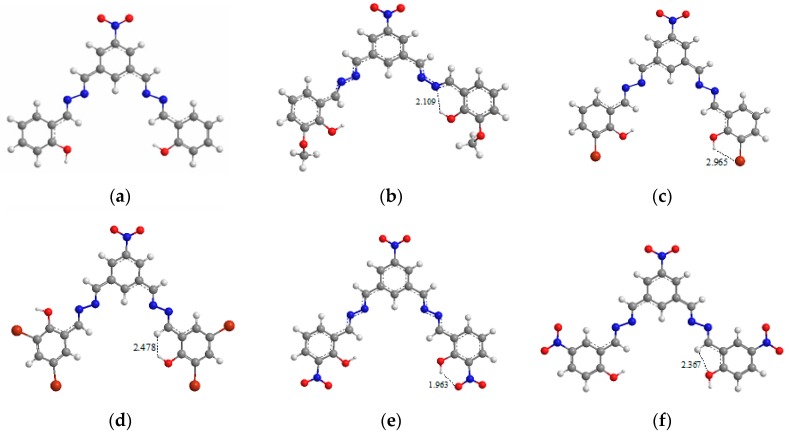
Optimized structures of six compounds. (**a**) Compound **1**; (**b**) Compound **2**; (**c**) Compound **3**; (**d**) Compound **4**; (**e**) Compound **5**; (**f**) Compound **6**.

To further understand the relationship between the structures and the photo-physical properties, the geometries of six compounds were optimized (Figure 6) using density functional theory at the B3LYP/3-21G level with the Gaussian03 program [44]. As shown in Figure 6, the six compounds all showed tripodal geometries. A different form of intramolecular hydrogen bond existed between the oxygen atom of −OH and the near-hydrogen atom for six compounds. The difference in intramolecular hydrogen bond induced a different red-shift effect in the UV-Vis spectra and different fluorescence responses of the host-guest.

**Figure 7:**
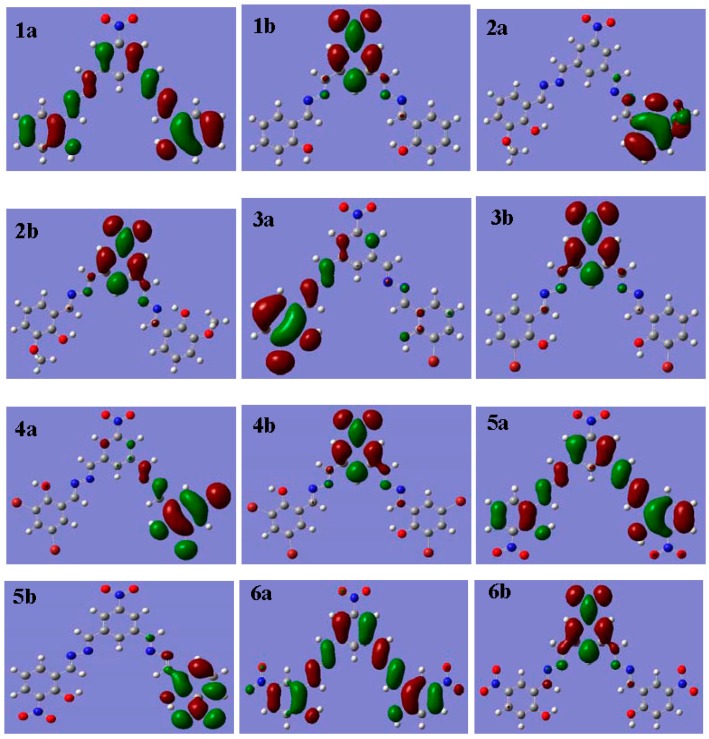
Selected HOMO (**a**) and LUMO (**b**) distributions of **1**–**6**. (**1a,b**) Compound **1**; (**2a,b**) Compound **2**; (**3a,b**) Compound **3**; (**4a,b**) Compound **4**; (**5a,b**) Compound **5**; (**6a,b**) Compound **6**.

The selected frontier orbitals for six compounds **1**–**6** were shown in Figure 7. The molecular frontier orbital was introduced in order to explain the UV-Vis absorption spectra in the host–guest interacted process by electron transition of the frontier orbital. Orbital analysis revealed that the highest occupied molecular orbital (HOMO) density in compounds **1**–**6** was localized mainly on the phenol moiety or on the whole molecule, whereas the lowest unoccupied molecular orbital (LUMO) density was localized on the nitrophenyl moiety. The electron transition of the lowest LUMO caused the red-shift phenomenon in the UV-Vis spectra.

## 4. Conclusions

Six tripodal compounds were synthesized and demonstrated a highly sensitive and selective absorption assay for oxy-anions. Nanomaterials of three compounds **2**, **3**, **4** were developed and the SEM images were related to the space configuration. Although two compounds **3** and **4** had the same substituent (-Br), SEM images were significantly different because of the different numbers of bromine groups. Compound **6** that contained *m*-NO_2_ exhibited the strongest binding ability for AcO^−^ ions among the six compounds. The host–guest interaction accompanied by color changes may be used as a colorimetric probe for the AcO^−^ ion detection. This understanding of the AcO^−^ sensing mechanism helps to determine possible structural modifications and achieve new nanomaterials that have an acetate sensing capacity in aqueous solution. The results are useful in expanded applications of tripodal structure derivatives.

## Figures and Tables

**Table 1:** Binding constants of compounds with various anions.

Anions ^a^	AcO^−^	H_2_PO_4_^−^	F^−^	Cl^−^ (Br^−^, I^−^)
*K*_s_ (**1**)	(9.56 ± 0.05) × 10^3^	(4.11 ± 0.14) × 10^2^	(2.31 ± 0.03) × 10^4^	ND ^b^
*K*_s_ (**2**)	(1.28 ± 0.06) × 10^3^	(1.18 ± 0.01) × 10^3^	(2.45 ± 0.04) × 10^4^	ND
*K*_s_ (**3**)	(2.76 ± 0.03) × 10^4^	(2.33 ± 0.02) × 10^3^	(1.60 ± 0.04) × 10^4^	ND
*K*_s_ (**4**)	(5.65 ± 0.08) × 10^4^	(4.35 ± 0.02) × 10^4^	(4.10 ± 0.10) × 10^4^	ND
*K*_s_ (**5**)	(6.48 ± 0.11) × 10^4^	(5.67 ± 0.08) × 10^4^	(3.35 ± 0.05) × 10^4^	ND
*K*_s_ (**6**)	(8.49 ± 0.19) × 10^4^	(6.07 ± 0.07) × 10^4^	(4.69 ± 0.12) × 10^4^	ND

^a^ All anions were added in the form of tetra-*n*-butylammonium (TBA) salts; ^b^ The binding constant could not be determined.

## References

[1] González Mdel C., Otón F., Espinosa A., Tárraga A., Molina P. (2015). Tris (triazole) tripodal receptors as selective probes for citrate anion recognition and multichannel transition and heavy metal cation sensing. Org. Biomol. Chem..

[2] Velu R., Ramakrishnan V.T., Ramamurthy P. (2011). Selective fluoride ion recognition by a thiourea based receptor linked acridinedione functionalized gold nanoparticles. J. Photoch. Photobiolo. A Chem..

[3] Kim S.-H., Hwang I.-J., Gwon S.-Y., Burkinshaw S.M., Son Y.-A. (2011). An anion sensor based on the displacement of 2,6-dichlorophenol-indo-*o*-cresol sodium salt from a water-soluble tetrasulfonated calix[4]arene. Dyes Pigment..

[4] Yang C., Wu B., Chen Y., Zhang K. (2015). Gels based on anion recognition between triurea receptor and phosphate anion. Macromol. Rapid Commun..

[5] Cametti M., Rissanen K. (2013). Highlights on contemporary recognition and sensing of fluoride anion in solution and in the solid state. Chem. Soc. Rev..

[6] Bodo E., Ciavardini A., Dalla Cort A., Giannicchi I., Yafteh Mihan F., Fornarini S., Vasile S., Scuderi D., Piccirillo S. (2014). Anion recognition by uranyl-salophen derivatives as probed by infrared multiple photon dissociation spectroscopy and *ab initio* modeling. Chemistry.

[7] Shang X.F., Wang Y.L., Wei X.F., Fu Z.Y., Zhang J.L., Xu X.F. (2013). Synthesis and binding ability of molecular probes based on a phenanthroline derivative: Theory and experiment. Molecules.

[8] Lin Q., Cai Y., Li Q., Shi B.B., Yao H., Zhang Y.M., Wei T.B. (2015). Fluorescent “turn-on” detecting CN^−^ by nucleophilic addition induced schiff-base hydrolysis. Spectrochim. Acta A Mol. Biomol. Spectrosc..

[9] Cornes S.P., Davies C.H., Blyghton D., Sambrook M.R., Beer P.D. (2015). Contrasting anion recognition behaviour exhibited by halogen and hydrogen bonding rotaxane hosts. Org. Biomol. Chem..

[10] Gale P.A. (2003). Anion and ion-pair receptor chemistry: highlights from 2000 to 2001. Coord. Chem. Rev..

[11] Robinson S.W., Mustoe C.L., White N.G., Brown A., Thompson A.L., Kennepohl P., Beer P.D. (2015). Evidence for halogen bond covalency in acyclic and interlocked halogen-bonding receptor anion recognition. J. Am. Chem. Soc..

[12] Xu Z., Kim S.K., Yoon J. (2010). Revisit to imidazolium receptors for the recognition of anions: Highlighted research during 2006–2009. Chem. Soc. Rev..

[13] Tomich J.M., Wallace D., Henderson K., Mitchell K.E., Radke G., Brandt T., Amber C.A., Scott A., Ganthan J., Sullivan L. (1998). Aqueous solubilization of transmembrane peptide sequences with retention of membrane insertion and function. Biophys. J..

[14] Hossain M.A., Llinares J.M., Powell D., Bowman-James K. (2001). Multiple hydrogen bond stabilization of a sandwich complex of sulfate between two macrocyclic tetraamides. Inorg. Chem..

[15] Szumna A., Jurczak J. (2001). A new macrocyclic polylactam-type neutral receptor for anions—Structural aspects of anion recognition. Eur. J. Org. Chem..

[16] Beer P.D., Szemes F., Balzani V., Salà C.M., Drew M.G.B., Dent S.W., Maestri M. (1997). Anion selective recognition and sensing by novel macrocyclic rransition metal receptor systems. ^1^H-NMR, electrochemical, and photophysical investigations. J. Am. Chem. Soc..

[17] Sessler J.L., Cho D.G., Lynch V. (2006). Diindolylquinoxalines: Effective indole-based receptors for phosphate anion. J. Am. Chem. Soc..

[18] Furman P.A., Fyfe J.A., Clair St M.H., Weinhold K., Rideout J.L., Freeman G.A. (1986). Phosphorylation of 3’-azido-3’-deoxythymidine and selective interaction of the 5’-triphosphate with human immunodeficiency virus reverse transcriptase. Proc. Natl. Acad. Sci. USA.

[19] Král V., Sessler J.L. (1995). Molecular recognition via base-pairing and phosphate chelation. Ditopic and tritopic sapphyrin-based receptors for the recognition and transport of nucleotide monophosphates. Tetrahedron.

[20] Ojida A., Mito-oka Y., Sada K., Hamachi I. (2004). Molecular recognition and fluorescence sensing of monophosphorylated peptides in aqueous solution by bis (zinc (II)-dipicolylamine)-based artificial receptors. J. Am. Chem. Soc..

[21] Gale P.A. (2005). A phenylhydrazone-based indole receptor for sensing acetate. Chem. Commun..

[22] Lee G.W., Singh N., Jang D.O. (2008). Benzimidazole and thiourea conjugated fluorescent hybrid receptor for selective recognition of PO_4_^3−^. Tetrahedron Lett..

[23] Shao J., Lin H., Shang X.F., Chen H.M., Lin H.K. (2007). A novel neutral receptor for selective recognition of H_2_PO_4_^−^. J. Incl. Phenom. Macrocycl. Chem..

[24] Jimenez Blanco J.L., Bootello P., Benito J.M., Ortiz Mellet C., Garcia Fernandez J.M. (2006). Urea-, thiourea-, and guanidine-linked glycooligomers as phosphate binders in water. J. Org. Chem..

[25] Gunnlaugsson T., Glynn M., Tocci G.M., Kruger P.E., Pfeffer F.M. (2006). Anion recognition and sensing in organic and aqueous media using luminescent and colorimetric sensors. Coord. Chem. Rev..

[26] Feng C., Dai S., Wang L. (2014). Optical aptasensors for quantitative detection of small biomolecules: A review. Biosens. Bioelectron..

[27] Amendola V., Esteban-Gomez D., Fabbrizzi L., Licchelli M. (2006). What anions do to NH-containing receptors. Acc. Chem. Res..

[28] Jung H.S., Chen X., Kim J.S., Yoon J. (2013). Recent progress in luminescent and colorimetric chemosensors for detection of thiols. Chem. Soc. Rev..

[29] Nishiyabu R., Anzenbacher P. (2006). 1,3-Indane-based chromogenic calixpyrroles with push-pull chromophores: Synthesis and anion sensing. Org. Lett..

[30] Wang Y.H., Lin H., Shao J., Cai Z.S., Lin H.K. (2008). A phenylhydrazone-based indole receptor for sensing acetate. Talanta.

[31] Liu Z.Q., Shi M., Li F.Y., Fang Q., Yi T., Huang C.H. (2005). Highly selective two-photon chemosensors for fluoride derived from organic boranes. Org. Lett..

[32] Lin Z.H., Ou S.J., Duan C.Y., Zhang B.G., Bai Z.P. (2006). Naked-eye detection of fluoride ion in water: A remarkably selective easy-to-prepare test paper. Chem. Commun..

[33] Lin Z.H., Zhao Y.G., Duan C.Y., Zhang B.G., Bai Z.P. (2006). A highly selective chromo- and fluorogenic dual responding fluoride sensor: Naked-eye detection of F^−^ ion in natural water via a test paper. Dalton Trans..

[34] Xu Z., Kim S., Lee K.H., Yoon J. (2007). A highly selective fluorescent chemosensor for dihydrogen phosphate via unique excimer formation and PET mechanism. Tetrahedron Lett..

[35] Kumar G.S., Neckers D.C. (1989). Photochemistry of azobenzene-containing polymers. Chem. Rev..

[36] Harada J., Fujiwara T., Ogawa K. (2007). Crucial role of fluorescence in the solid-state thermochromism of salicylideneanilines. J. Am. Chem. Soc..

[37] Liu Y., Han B.H., Zhang H.Y. (2004). Spectroscopic studies on molecular recognition of modified cyclodextrins. Curr. Org. Chem..

[38] Liu Y., You C.C., Zhang H.Y. (2001). Supramolecular Chemistry.

[39] Bourson J., Pouget J., Valeur B. (1993). Ion-responsive fluorescent compounds. 4. Effect of cation binding on the photophysical properties of a coumarin linked to monoaza-and diaza-crown ethers. J. Phys. Chem..

[40] Bonizzoni M., Fabbrizzi L., Taglietti A., Tiengo F. (2006). (Benzylideneamino) thioureas—Chromogenic interactions with anions and N-H deprotonation. Eur. J. Org. Chem..

[41] Frisch M.J., Trucks G.W., Schlegel H.B., Frisch M.J., Trucks G.W., Schlegel H.B., Scuseria G.E., Robb M.A., Cheeseman J.R., Montgomery J.A. (2003). Software for Computational Chemistry.

[42] Smith K., Musson A., De Boos G. (1996). Superior methodology for the nitrate ion of simple aromatic compounds. Chem. Soc. Chem. Commun..

[43] Zhang X., Zhang X., Zou K., Lee C.S., Lee S.T. (2007). Single-crystal nanoribbons, nanotubes, and nanowires from intramolecular charge-transfer organic molecules. J. Am. Chem. Soc..

[44] Hu D.H., Yu J., Padmanadan G., Ramakrishanan S., Barbara P.F. (2002). Spatial confinement of excition transfer and the role of conformational order in organic nanoparticles. Nano Lett..

